# Will cities keep getting hotter? The interplay of urban expansion and greening reshapes future urban heat trajectories

**DOI:** 10.1093/pnasnexus/pgag070

**Published:** 2026-03-16

**Authors:** Huidong Li, Lin Meng, Hiba Baroud, T C Chakraborty, Jiafu Mao, Zhonghua Zheng

**Affiliations:** Department of Earth and Environmental Sciences, Vanderbilt University, 5726 Stevenson Science Center, Nashville, TN 37235, USA; Department of Earth and Environmental Sciences, Vanderbilt University, 5726 Stevenson Science Center, Nashville, TN 37235, USA; Department of Civil and Environmental Engineering, Vanderbilt University, PMB 351831 2301 Vanderbilt Place, Nashville, TN 37235, USA; Atmospheric Sciences and Global Change Division, Pacific Northwest National Laboratory, 902 Battelle Boulevard, Richland, WA 99352, USA; Environmental Sciences Division, Oak Ridge National Laboratory, 1 Bethel Valley Road, Oak Ridge, TN 37830, USA; Department of Earth and Environmental Sciences, The University of Manchester, Oxford Rd, Manchester M13 9PL, United Kingdom

**Keywords:** urbanization, greening initiative, urban heat island profile, urban heat mitigation

## Abstract

Urban heat islands (UHIs) pose growing risks to public health, infrastructure, and resilience. While often assumed to intensify with urban growth, dynamic changes in urban expansion and vegetation greenness complicate UHI trajectories, which remain poorly understood. This study investigated the interplay of urban expansion and greenness change on UHI spatial profile across 36 Chinese megacities during 2003–2018 using multiple satellite products. We introduce a framework that classifies urban areas into four dynamic development pathways based on impervious surface area (ISA) and enhanced vegetation index (EVI) trends: urbanized-greening, urbanized-browning, urbanizing-greening (UingG), and urbanizing-browning (UingB). While most urbanized centers exhibited greening driven by targeted initiatives and urbanizing suburbs showed browning due to vegetation loss, about 30% urban areas showed the reverse pattern, revealing overlooked complexity in urban development. Urban expansion and browning strengthened UHI in suburban areas, whereas greening initiatives mitigated UHI in urban center and mitigated UHI enhancement in suburban areas. Slowed warming in urban centers together with accelerated warming in suburban areas flattened the temperature gradient between urban centers and suburbs. This dynamic expanded the spatial extent of elevated temperatures and reshaped the classic urban-to-rural UHI profile into a flatter form. In UingB areas, UHI intensification was jointly driven by increase in ISA, vegetation loss, and their interaction, while in UingG areas, EVI increases and a negative interaction together offset over half of the warming driven by urban expansion. These findings reveal that UHI evolution is not unidirectional but depends on localized urbanization and greening dynamics, offering pathways for strategic heat mitigation.

Significance Statement Urban heat islands (UHIs) are often assumed to intensify indefinitely as cities grow. However, we show that the future of urban heat is more dynamic and depends critically on how urban expansion and vegetation changes interact. We find that urban greening can slow or even reverse UHI intensification in city centers, while suburban expansion together with vegetation loss exacerbates heat risks. These findings challenge the traditional view of UHI evolution and reveal that targeted greening efforts in both urban cores and suburban areas are essential for mitigating extreme heat risks. Our results offer actionable insights for sustainable urban development, emphasizing the need to integrate vegetation management into urban planning to build more resilient cities.

## Introduction

Continuous urban expansion globally has substantially modified regional climate in cities, leading to higher temperatures than adjacent rural areas, namely the urban heat island (UHI) effect (1). These localized warming effects are further exacerbated by global climate change, increasing heat risks in urban areas (2–4), with severe implications for public health, energy consumption, and air and water quality (5–7). In response to rising heat stress, urban greening initiatives, such as tree planting and green roofs, have emerged as effective strategies to mitigate UHI effect. Studies show that green spaces can reduce local temperatures by 10 °C in summer (8–12). As a nature-based solution, urban greening has been widely adopted to enhance resilience and improve overall urban sustainability (13–15).

However, urbanization-driven warming and greening-driven cooling often occur simultaneously within cities, with rates and intensities varying across neighborhoods (16). This complexity challenges the traditional understanding of UHI spatial profiles (ie spatial pattern of UHI along urban–rural gradients), which are commonly characterized as steep temperature gradients from hotter urban cores to cooler rural surroundings, primarily driven by increased impervious surfaces and vegetation loss in urban centers (17, 18). In reality, UHI spatial and temporal dynamics are far more heterogeneous, shaped by complex and interacting land surface features and their evolution (19, 20). Suburban areas undergoing continuous urbanization often experience impervious surface expansion and vegetation loss (21, 22), thereby intensifying UHI effects (23–25). In contrast, urban cores often benefit from greening initiatives that contribute to localized cooling, particularly from 3D tree canopies compared to low-lying vegetation (19). These contrasting trends can reshape the UHI profile, shifting cities away from traditional triangular UHI profiles (ie temperatures peak in dense urban centers and decline toward cooler suburban and rural areas) toward trapezoid, concave-centered, or multicentered UHI profiles, where suburban areas become emerging heat hotspots (26–28). Understanding these interactive dynamics is critical for developing targeted heat adaptation and mitigation strategies.

Despite their importance, most urban studies have focused primarily on a single process, either UHI response to urban expansion or the cooling efficiency of urban greening, neglecting the temporal interactions between urbanization and greenness change across different areas of cities (25, 29). Few have examined how concurrent urbanization and greening pathways collectively shape UHI profile over time, often assuming these processes are mutually exclusive, with urbanization always resulting in green space loss (30, 31). Many analyses overlook cases where greening, such as tree planting, occurs alongside urban development in fragmented vacant lands (32, 33). Studies based on land-use classifications have traditionally failed to capture continuous subgrid changes in imperviousness and greenness that do not alter land-use types (34, 35). As a result, studies that examine only urban expansion tend to underestimate its impact on UHI, while those focusing solely on greening often underestimate its cooling effects. Moreover, urban–rural dichotomy approaches or “space-for-time” substitutions, commonly used to analyze UHI intensity in relation to urban expansion and greening spatially, failed to account for evolution of UHI profile over time (36, 37), therefore obscuring the interactive impacts of concurrent urbanization and greening on UHI profiles (38–41). Consequently, the question of how and where the UHI profile changes within a city remains an open debate.

Chinese cities serve as highly representative cases that experienced concurrent urban expansion and greening over the past several decades. Early phases of China's urbanization prioritized infrastructure development, often replacing natural vegetation and farmland with buildings and roads (42–44). In recent decade, efforts to expand green infrastructure have intensified, particularly in highly urbanized areas (45–48). Since 2004, urban vegetation trends have shifted from decline to recovery (49–51), which proved to be effective in mitigating UHI effect (38, 52). Greening trends decreased from urban cores toward newly developed suburban towns and then transitioned to browning at urban fringes (21). These diverse urbanization pathways provide a unique opportunity to examine the interactive effects of urban expansion and greening on UHI profile.

The goal of this study is to quantify the varying concurrent urbanization and greening dynamics and their interactive impact on daytime surface UHI profile in 36 major cities in China during 2003–2018. We answer three research questions: (i) what are the relative proportions of different urbanization-greening dynamics in these cities? (ii) How do the various urbanization and greening dynamics drive the spatiotemporal evolution of UHI profiles? (iii) What are the respective contributions of urbanization and greening to changing UHI? To answer these questions, we classified urban areas within each city into four distinct dynamic categories based on the trends of urbanization (as defined by the impervious surface area [ISA]) and greenness (as defined by the enhanced vegetation index [EVI]) over time: urbanized-greening (UedG), urbanized-browning (UedB), urbanizing-greening (UingG), and urbanizing-browning (UingB) (see Methods). Surrounding rural areas of the same size were used as baselines to isolate the impacts of climate change in quantifying UHI intensity. We quantified the changes in EVI and ISA and examined their interactive impacts on UHI profile in each of the four urban dynamic categories. To better quantify long-term evolution of UHI across all seasons and overcome the short-term disturbance due to weather change, we applied an annual temperature cycle (ATC) model to moderate resolution imaging spectroradiometer (MODIS) land surface temperature (LST) data. This model calculates key parameters, including the annual mean temperature (*C_m_*) and amplitude (ie difference between max and min, *C_a_*), and the date of the hottest day (*C_p_*), generating a continuous daily temperature time series for each pixel within each city (53). Ultimately, this research contributes to understanding the temporal evolution in UHI profile with urban expansion and greenness change, providing a robust scientific foundation for urban planning for resilient cities.

## Results

### Divergent urban development pathways

**Fig. 1.**
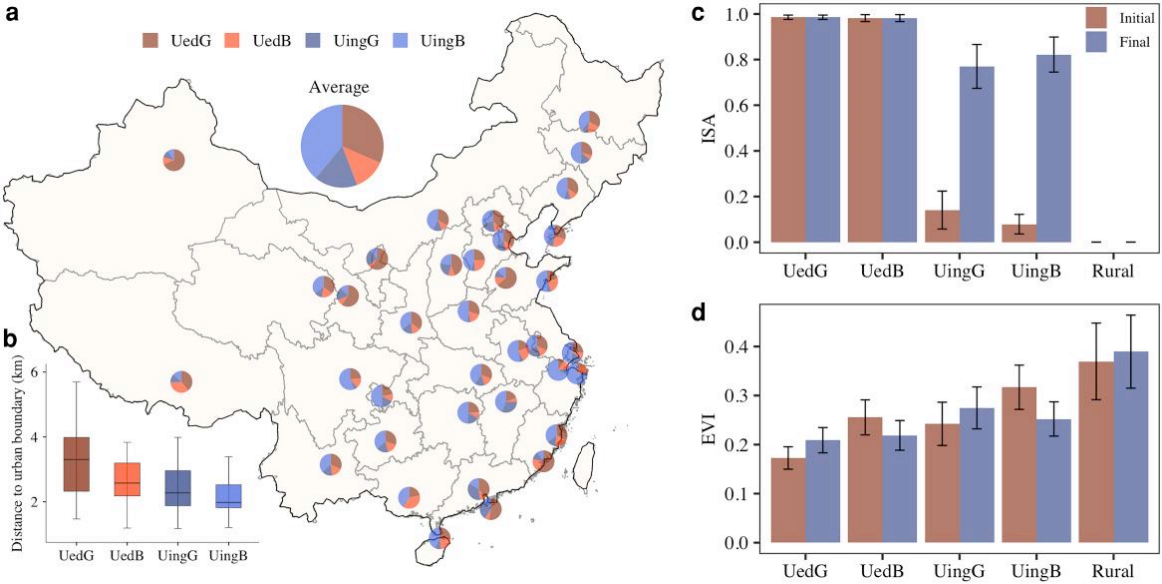
Divergent urban development pathways across all study cities. (a, b) Spatial pattern of area percentages of four urban dynamic categories in 36 cities and their distance to urban boundary. (c, d) Mean ISA and EVI between initial years (2003–2005) and final years (2016–2018). Error bar represents 1 SD among cities. UedG, UedB, UingG, and UingB.

All the cities considered here present divergent development pathways during 2003–2018 with four urban dynamic categories, driven by varying rates of urban expansion and greening initiatives. On average across all cities, UingB and UedG occupy the largest percentage of urban areas (39 and 32%, respectively), followed by UingG (17%) and UedB (13%) (Fig. 1a). This highlights prevalent vegetation loss or degradation associated with urban expansion in suburbs, accompanied by concurrent greening initiatives in densely urbanized areas. Notably, however, 30% of urban areas—a substantial proportion—experience either urban expansion alongside greening (UingG) or browning within already urbanized regions (UedB). This dynamic, which has received limited attention in prior studies, underscores the complexity of how urbanization interacts with localized greening and browning processes.

The proportions of these urban dynamic categories vary largely across cities (Fig. S1). Urbanizing areas (UingG + UingB) occupy more than half of urban areas in 62% of study cities, indicating rapid urbanization during the study period. For example, Hangzhou, a fast-developing city, has the highest percentage of urbanizing areas (77% of urban areas), comprising 66% UingB and 11% UingG (Fig. S2). Greening areas (UedG + UingG) exceed half of urban areas in over one-third of the cities, and 84% of cities have at least 30% of urban areas with greening trend, reflecting widespread greening initiatives. For example, Beijing and Shanghai have 38 and 25% urban areas with greening trends in urbanized areas (UedG), whereas 23 and 20% in urbanizing areas (UingG), respectively. Even arid cities in northwest region contain large UedG areas, reflecting the implementation of effective greening initiatives.

Generally, UedG and UedB, located in the urban center, exhibit low EVI and high ISA, whereas UingG and UingB, located in suburban areas, start with low ISA and high EVI in initial period (Fig. 1b–d). These spatial differences in EVI and ISA across the four categories were pronounced in 2003 but diminished over the years. ISA remained consistently high in fully urbanized areas (mean >0.98) with minimal variation over time, whereas it increased significantly (*P* < 0.01) in urbanizing areas (Fig. S3a). Specifically, ISA rose from 0.08 to 0.80 in UingG and from 0.04 to 0.84 in UingB, reflecting ongoing urban expansion. At the same time, EVI increased in UedG and UingG (0.03/decade each, *P* < 0.01), faster than rural greening induced by climate change, demonstrating the effect of urban greening initiatives (Fig. S3b). In contrast, EVI decreased in UedB (−0.03/decade) and UingB (−0.05/decade). Across cities, ISA and its changes show minimal spatial variation in suburbs, reflecting a consistent national pattern of urban expansion (Fig. S4a and b). Similarly, urban EVI and its trends exhibit lower spatial variability compared to those observed in rural EVI (Fig. S4c and d). This highlights the influence of extensive urban greening initiatives in enhancing urban EVI, effectively offsetting the variability in rural EVI that is primarily driven by climatic changes.

### Changes in annual UHI cycle

**Fig. 2.**
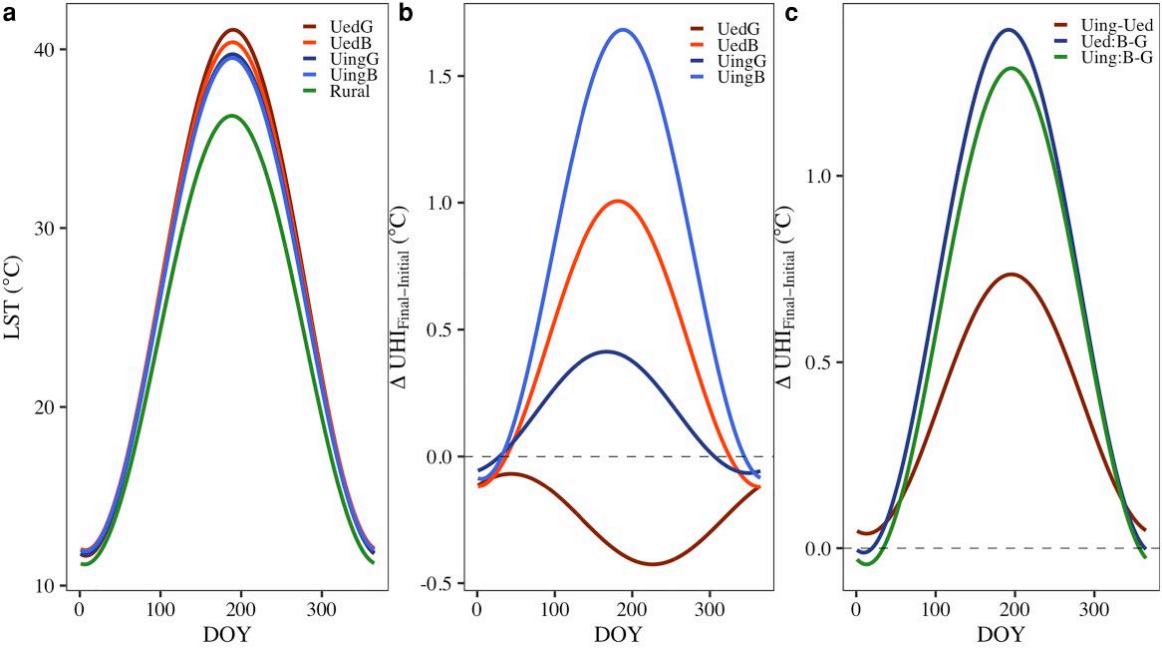
Annual cycles of UHI and their change across all study cities. (a) Mean annual LST cycle during 2003–2018; (b, c) Changes of annual UHI cycle (b) and their spatial difference (c) between initial (2003–2015) and final (2016–2018) periods. Dashed black lines represent y = 0. The color represents different urban dynamic categories and their spatial difference: UedG, UedB, UingG, UingB. UedB–UedG (Ued:B–G), and UingB–UingG (Uing:B–G).

Alongside the variations in ISA and EVI, the ATC patterns exhibited differences in both magnitude and seasonal shifts across the four urban dynamic categories (Fig. 2a). All four urban categories were warmer than rural areas throughout the year, indicating distinct UHI effects. On average, UedG had the highest annual mean temperature (*C_m_*) and amplitude (*C_a_*), followed by UedB, UingG, and UingB (Fig. S5a and b), indicating the decreasing UHI along the urbanization levels. Driven by the changes in EVI and ISA between 2003 and 2018, the urban–rural difference of ATC (ie annual UHI cycle) patterns changed variedly among four urban dynamic categories. The urban–rural differences in annual mean temperature (Δ*C_m_*) and amplitude (Δ*C_a_*) increased notably in UingB, UedB, and UingG, while declining in UedG (Fig. S5c and d). As a result, annual UHI generally increased in UingB, UedB, and UingG, but decreased in UedG (Figs. 2b and S6a), implying urban expansion in UingD and UingG and vegetation degradation in UedB amplified UHI, while the greening in UedG mitigated UHI. Meanwhile, the compensatory cooling effect of greening substantially reduced the enhancement of UHI in UingG during urban expansion, leading to a smaller UHI increase in UingG than in UingB. Seasonally, both UHI and its change were the largest in summer and the least in winter. Overall, UHI in urbanizing areas increased much more than in urbanized areas (Fig. 2c), reaching 0.65 °C in summer and accumulating to 252.05 °C•day annually. Meanwhile, due to the influence of opposite greenness changes in the subcategories (ie cooling in greening areas and warming in browning areas), UHI difference between them decreased from 2003 to 2018 (Figs. 2c and S6b). Such decrease in urbanized areas reached 1.39 °C in summer and accumulated to 252.05 °C•day annually, much more than that in urbanizing areas (1.28 °C in summer and 227.45 °C•day annually). In the following analysis, we primarily focus on summer UHI profile and its response to urban expansion and greening, given its substantial variations across four categories and its critical relevance to heat waves.

### Expanded but flattened UHI

**Fig. 3.**
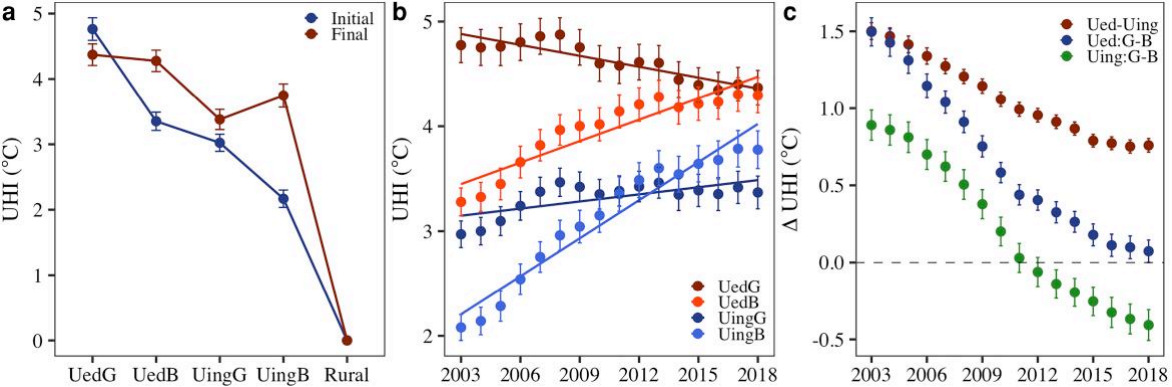
Expanded summer UHI extent with flattened gradients. (a) Temporal change of UHI; (b) change in UHI profile between initial (2003–2015) and final (2016–2018) years; (c) temporal change in spatial difference of UHI. Error bar represents one-tenth of the SD among cities. ΔUHI was calculated by subtracting values in browning from greening regions. UedG, UedB, UingG, UingB, UedG–UedB (Ued:G–B), and UingG–UingB (Uing:G–B).

The varying warming rates among the four urban dynamic categories resulted in an expanded extent but flattened gradient of the summer UHI profile—indicating larger heated areas but reduced temperature contrasts from urban core to suburban areas (Fig. 3). In the initial years, there was a sharp decline of temperature along the urban–rural gradient (Fig. S7), with the highest UHI intensity in UedG (4.75 °C), followed by UedB (3.34 °C), UingG (3.01 °C), and UingB (2.16 °C) (Fig. 3a). Subsequently, UHI experienced significant (*P* < 0.01) intensification in urbanizing areas compared to urbanized areas, and in browning areas compared to greening areas (Fig. 3b). Over years, UingB showed the largest UHI increase (0.98 °C/decade) due to the combined effect of urban expansion and browning. Strong browning also contributed to a notable UHI increase in UedB (0.58 °C/decade), despite no urban expansion. In contrast, affected by cooling effect of greening, UingG exhibited only a slight increase in UHI (0.22 °C/decade), which was substantially smaller than the increase observed in UingB, despite both experiencing similar levels of urban expansion. UedG even experienced a decrease in UHI (−0.24 °C/decade), due to the strong cooling effect of greening. As a result, UHI difference between urbanized and urbanizing areas, representing the impact of urban expansion, decreased from 1.50 °C to 0.75 °C, indicating reduced UHI variation across city over time (Fig. 3c). At the same time, UHI difference between greening and browning subcategories also decreased. Specifically, UHI difference between UedG and UedB declined from 1.49 °C to 0.07 °C, rendering UHI nearly identical in these two subcategories in the final period. UHI difference between UingG and UingB markedly decreased from 0.89 °C to −0.41 °C. Consequently, by the end of study period, although the temperatures increased across the city (Fig. S7), UHI profile became more flattened along the urban–rural gradient (Fig. 3a).

### Attribution between ISA, EVI, and their interactions

**Fig. 4.**
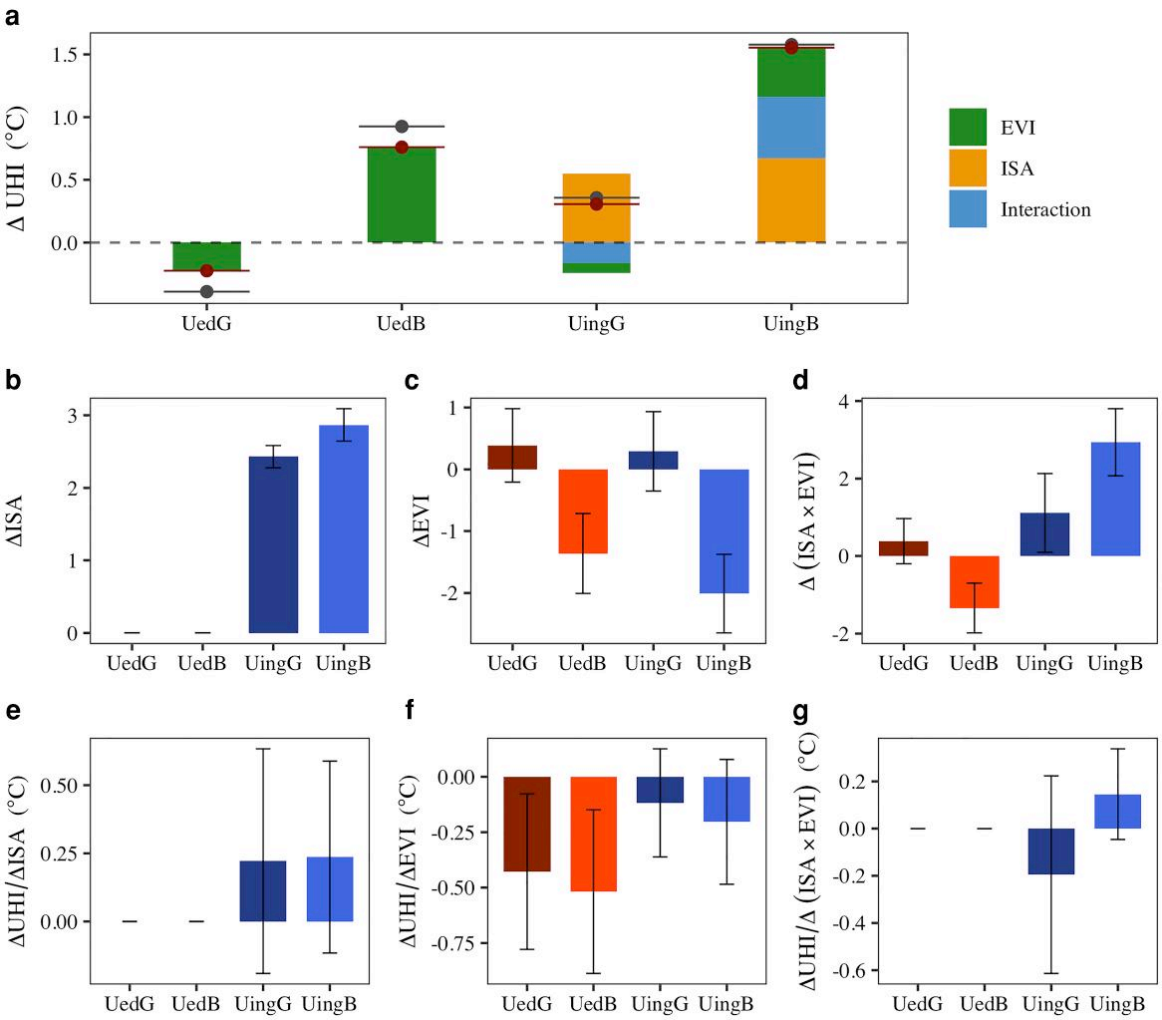
Urban expansion amplifies while greening mitigates summer UHI changes. (a) Attribution of ISA, EVI, and their interactions to UHI changes across four urban dynamic categories in all cities during 2003–2018. Gray and red points with horizontal lines represent the observed and modeled net changes in UHI, respectively, after accounting for the offsetting effects of ISA, EVI, and their interaction (ISA × EVI). (b–d) The changes of ISA, EVI, and ISA × EVI. (e, f) the response of UHI to the changes in ISA, EVI, and ISA × EVI. The changes of EVI, ISA, and ISA × EVI were calculated using the mean values between initial period (2003–2005) and final period (2016–2018). ISA, EVI, and ISA × EVI were normalized before analysis to ensure comparability of effect sizes, while UHI was retained in its original units. ΔUHI/ΔISA, ΔUHI/ΔEVI, and ΔUHI/Δ(ISA × EVI) were determined by calculating the slopes of multiple linear regression models for each urban dynamic category and each city (see Methods). Error bar represents 1 SD among cities.

To better understand the respective impact of urban expansion and greenness change on the change of UHI profile, we disentangled the contributions of ISA, EVI, and their interactions to summer UHI change (ΔUHI_ISA_ and ΔUHI_EVI_) during 2003–2018 in each of the four urban dynamic categories (Fig. 4a), using the multiple linear regression models with the interaction term (see Methods). We found that UHI responses were not simply additive, ie changes in EVI modulated the thermal effect of urban expansion, amplifying or offsetting warming depending on EVI trends. In urbanized areas with minimal urban expansion, ΔUHI was solely caused by vegetation changes, whereas in urbanizing areas, ΔUHI was jointly driven by urban expansion and vegetation change as well as their interaction (Fig. S8). In UingB, the increase in ISA contributed to 43.2%, the decrease in EVI led to 25.4%, and the interaction led to 31.3% increase in UHI. Positive interaction effect indicates that as ISA increases (urban expansion) and EVI decreases (vegetation loss) occur together, the combined warming impact on UHI is more than expected from adding each effect separately. In UingG, the ISA increase led to a 178.6% increase in UHI, but the increase in EVI offsetting the enhancement of UHI by 25.9% while interaction offsetting UHI by 52.7%. Negative interaction shows that when both ISA and EVI increase (vegetation gain during urbanization), the cooling influence of greening further offsets the ISA warming.

The contributions of ISA and EVI on UHI were determined not only by the absolute change of ISA and EVI themselves (Fig. 4b–d) but also by the UHI response to their changes, ie ΔUHI/ΔISA, ΔUHI/ΔEVI, and ΔUHI/Δ(ISA × EVI) (Fig. 4e–g). In urbanizing areas, the increase in ISA was larger in UingB compared to UingG, although ΔUHI/ΔISA were similar, leading to a slightly larger contribution of ΔISA on ΔUHI in UingB. Regarding changes in greenness, ΔUHI exhibited a larger response to ΔEVI in urbanized areas than in urbanizing areas, demonstrating higher cooling and warming efficiencies in greening and browning subcategories, respectively. The former resulted in stronger cooling effects in urbanized areas compared to urbanizing areas, while the latter contributed to a reduced disparity in the warming effect between UedB and UingB, relative to their difference in EVI decline. The interaction term, ΔUHI/Δ(ISA × EVI), further revealed a negative sensitivity in UingG but positive sensitivity in UingB (Fig. 4g), suggesting that the interactive influence of ISA and EVI on UHI is subadditive, ie concurrent greening during urban expansion reduces the warming expected from ISA increases, while co-occurring browning and expansion amplify urban warming.

## Discussion

**Fig. 5.**
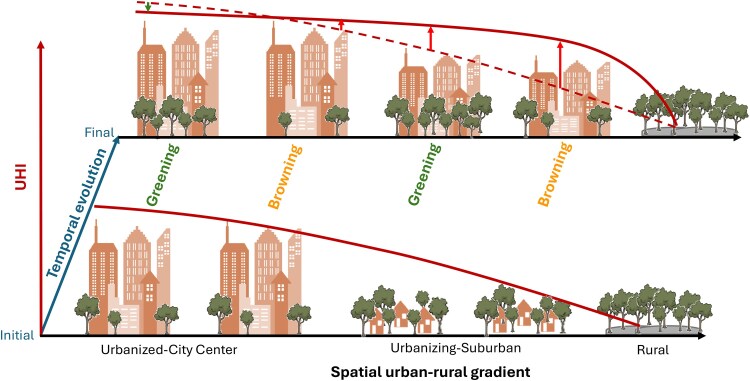
Schematic diagram of UHI profiles evolution driven by urban expansion and greenness change across the four urban categories. Urbanized greening areas exhibit a decline in UHI, while UedB, UingG, and UingB areas all experience an increase UHI, with the magnitude of increase escalating progressively from UedB to UedB. The dashed and solid red lines in final years represent UHI in initial years and final years for comparison. Vertical green and red arrow represents decline and increase in temperature in final years, respectively, compared to initial years.

This study revealed the interactive impacts of urban expansion and greenness change in shaping UHI profile through diverse development pathways within 36 megacities in China. We found urban expansion in suburban areas elevated temperature, decreasing the overall UHI difference between urbanized and urbanizing areas, whereas the greening in the urban center mitigated UHI. As a result, the UHI areas expanded but urban–rural gradient flattened, shifting from a sharp initial decline to a more gradual one (Fig. 5). As cities continue to face rising temperatures and urbanization, the study's insights carry critical implications of prioritizing greening initiatives in urban cores and fast-urbanizing areas to effectively mitigate UHI effects. Balancing infrastructure development with green space conservation is crucial for reducing heat risks, enhancing thermal comfort, and improving climate resilience, supporting sustainable urban growth in a changing climate (15, 54).

Our goal in defining the four urban categories was to create a simple yet conceptually clear framework that captures dominant patterns of coupled urbanization and vegetation change, revealing first-order, spatially consistent trajectories between urban expansion and greenness changes. Unlike traditional methods that primarily focus on urban expansion characterized by discrete land-use changes (55, 56), our approach captures gradual shifts in both imperviousness and greenness that may not alter land cover types but are critical for understanding subgrid dynamics often overlooked in both observation and models. Using this method, we identified areas with subtle yet meaningful vegetation degradation or greening, particularly in urbanized areas. Our study reveals that 30% of urban areas experienced simultaneous urban expansion and greening in suburban areas or browning in urban centers, which is previously underexplored in UHI research. These detailed patterns are instrumental in shaping UHI profiles, challenging the conventional assumption that urban expansion invariably leads to browning (30, 31). This categorization can be applied to refine UHI models by incorporating subgrid vegetation and impervious surface dynamics, enabling researchers and urban planners to better identify localized hotspots, assess the effectiveness of greening initiatives, and develop targeted mitigation strategies tailored to specific urban development patterns.

Several findings in this study challenge the common understanding of UHI dynamics. First, we found that UHI intensity does not continue to increase indefinitely in highly urbanized center areas. Instead, increases are primarily concentrated in suburban areas experiencing vegetation degradation and continuous urban expansion. Simultaneously, greening efforts in urban centers can reduce UHI intensity, highlighting that the future UHI trajectories will vary significantly depending on the extent and success of greening initiatives. Additionally, while previous studies have linked UHI intensity to urban expansion and a positive correlation with urban size (36, 37, 57), our findings reveal that the rate of UHI increase varies across different parts of a city. Although UHI-affected areas will expand, the temperature contrast between urban center and suburban areas will decrease, resulting in a flatter spatial gradient of UHI from urban to suburban regions, while maintaining a steeper gradient from suburban to rural areas. With robust greening efforts and ongoing suburban expansion, some cities may eventually shift to a trapezoid and even concave-centered UHI profile in the future, characterized by cooler urban cores and warmer suburban zones, contrasting with the current triangular UHI profile, where temperatures peak in urban center and decline outward (58). While the transition toward a flat UHI profiles is still underway, the observed trends underscore the need to account for evolving, rather than static, UHI profiles when modeling future trajectories.

The implications of these findings are profound for urban planning and climate adaptation strategies, particularly in the context of the inequitable distribution of the cooling benefits of trees within cities. While the cooling effects of urban vegetation are widely recognized, certain areas remain underserved. Our finding reveals protecting existing urban green space and increasing vegetation cover are essential actions to enhance the climate change resilience of urban areas. In urban centers, which often have much higher population densities than suburban and rural areas, residents are exposed to greater risks from heat waves and are more vulnerable to climate warming (26). Expanding green spaces in these densely populated areas is crucial for mitigating extreme heat risks and improving public health outcomes. Moreover, we found the stronger cooling efficiency of greening in highly urbanized areas compared to newly urbanizing areas, consistent with recent studies (59, 60), further underscoring the importance of prioritizing greening initiatives within densely built-up city centers. At the same time, a flattened UHI gradient from urban to suburban areas suggests that suburban areas, previously less affected by heat, are now experiencing greater heat intensification due to urban expansion. This highlights the urgent need for greening initiatives in newly urbanizing suburban areas to mitigate rising heat exposure, a factor often overlooked in urban planning (21).

Importantly, the feasibility of implementing such greening strategies will vary across cities due to differences in governance structures, fiscal capacities, spatial planning constraints, and climate zones. Large, well-resourced cities may be able to invest in ambitious greening programs, including large-scale park creation, vertical greening, and rooftop gardens, whereas smaller or less affluent cities may need to prioritize lower-cost measures such as community tree planting or protecting remnant green patches. Rapidly expanding informal settlements require flexible, incremental approaches distinct from those in mature urban cores. Likewise, greening strategies should be tailored to climate and development context. In semiarid cities, drought-tolerant planting and efficient irrigation are essential, while in humid subtropical regions, expanding tree canopy can deliver strong cooling benefits. Policymakers and city planners can leverage these insights to implement differentiated and context-specific interventions that reduce heat exposure across the urban–suburban–rural gradients.

While LST, as used in this study, is not directly equivalent to ambient heat exposure (more commonly associated with air temperature), it remains an important component of the urban thermal environment. LST strongly influences thermal comfort through its control of longwave radiation exchange between urban surfaces and the human body, as well as by affecting near-surface air temperature and mean radiant temperature. Thus, although LST is less direct than air temperature in representing human heat exposure, it captures critical aspects of surface energy balance that determine perceived heat stress. Nevertheless, differences between LST and air temperature can arise from surface heterogeneity, atmospheric mixing, and meteorological variability (61). Accordingly, we interpret our findings with caution: the directional trends in UHI dynamics observed here are likely to reflect corresponding changes in air temperature, but the absolute magnitudes of heat exposure may differ.

Additional limitations include our reliance on linear trend analyses, which may not capture nonlinear or abrupt changes in urban development or greenness, and the use of equal-area approach used to define rural baselines, which, although widely adopted in urban–rural comparison studies (37, 38, 41, 57), may not fully account for local elevation differences that influence temperature independently of urbanization. Finally, while our analysis covers 36 Chinese megacities, future research should extend to a broader set of cities across diverse climate zones, socioeconomic contexts, and urban forms to improve generalizability and to test how greening–urbanization interactions play out globally.

This study reveals that UHI dynamics are more complex than traditionally understood, with temperature profiles varying significantly across urban centers, suburban areas, and rural regions. Our findings challenge the assumption that urbanization uniformly leads to vegetation browning and intensified UHI effects. Instead, urban centers with greening initiatives exhibit a flattened, trapezoid, or even concave-centered UHI profile, where suburban areas may experience the highest temperatures. These findings highlight the critical role of vegetation dynamics in shaping UHI gradients and emphasize the necessity of targeted urban planning strategies that integrate greening efforts in both urban cores and suburban areas. This has critical implications for climate adaptation in densely populated urban centers, where limited vegetation and extensive impervious surfaces heighten exposure to intensifying heatwaves under a warming climate. Moving forward, integrating vegetation dynamics into UHI models and examining the socioeconomic benefits of urban greening can provide a more comprehensive framework for designing climate-resilient cities.

## Methods

### Datasets and processing

Open-accessed satellite daily LST, monthly EVI, yearly ISA, and yearly land cover type data were used in the study.

Daily LST data from MODIS product (MYD11A1) collection 6 were used to investigate the dynamic of UHI and the cooling effect of greening. The MODIS LST data provides daily per-pixel LST and emissivity with 1 km spatial resolution since 2003. It is derived using the generalized split-window algorithm, which effectively accounts for atmospheric interference to provide accurate surface temperature estimates. Following retrieval, additional bias correction processes further enhance the precision of the LST measurements. MODIS LST are known for their high accuracy, with temperature error margins typically within ±1 K under most conditions (62) and have been widely used to study urban heat islands regionally and globally (2, 28). This study mainly focused on the daytime LST observed at 1:30 PM local time when the maximum temperature, strong surface UHI and its spatiotemporal variation (40, 63), and cooling effect of green space (64, 65) occurred.

Monthly composite EVI data from MODIS product (MYD13A3) collection 6 at 1 km resolution were used to quantify greenness and the greening trend for each city. This product is derived from the Aqua satellite, leveraging near-infrared and red reflectance bands to capture vegetation health, productivity, and biomass. MYD13A3 is processed to minimize atmospheric and view angle effects, yielding high-quality, globally consistent vegetation metrics. These indices are widely used for assessing global vegetation dynamics and urban greenness change (21). We calculated the mean EVI in summer (June, July, and August) each year to quantify and explain the greenness change.

ISA data from annual maps of global artificial impervious area (GAIA) was used to quantify urbanization levels and dynamic. GAIA records the year of impervious area construction at a resolution of 30 m between 1985 and 2018 (22). Developed by integrating Landsat satellite imagery with machine learning algorithms, GAIA offers spatial detail, capturing annual changes in artificial surfaces, including roads, buildings, and other urban infrastructure. GAIA's detailed ISA data supports urban planning, climate resilience strategies, and ecosystem assessments on a global scale.

MODIS land cover type data (MCD12Q1) collection 6 at a resolution of 500 m in 2018 was used to determine the urban extent of each city. The MCD12Q1 product provides annual global land cover classification, derived from MODIS data on both the Terra and Aqua satellites. It includes 17 land cover types (eg forests, water, and urban areas). We used the pixels of “Urban and Built-up Lands” in the land cover data to determine the boundary of the city (see details in Urban dynamic categories section). Meanwhile, we excluded pixels of water bodies in all analysis.

Given the differing data lengths of MODIS products and GAIA ISA data, we chose the study period from 2003 to 2018. To ensure consistency across datasets with varying spatial resolutions and projections, all raster data were reprojected and resampled to match MODIS LST pixels at a 1 km resolution.

### Study cities

We investigated 36 representative cities across different geographical regions in Mainland China, including four municipalities, 27 provincial capital cities, and five cities specifically designated in the state plan. All these cities are the central cities nationally and regionally in terms of population and economy in China. They have experienced fast urbanization and played a leading role in the development of society and economy. The selected cities account for over one-third of China's total urban area and ∼45% of the national urban population in 2018, based on data from the 2018 China Urban Construction Statistical Yearbook (https://www.stats.gov.cn). Moreover, these cities continue to experience rapid development and expansion in alignment with the Chinese government's urbanization plan. At the same time, they implemented a series of greening initiatives to adapt to climate change and improve livability, including public greening, residential greening, and scenic greening, following the ordinance for urban greening designed by the government in 2001 (14, 43). Following these efforts, urban vegetation cover in these megacities saw a substantial increase after 2004 (49), with the greening rate nearly twice that of smaller cities (48). Notably, these anthropogenic greening measures substantially enhanced vegetation cover even in semiarid and arid cities in northwestern regions, effectively reducing the disparities in greenness among cities driven by climatic differences (42, 50).

### Urban dynamic categories

We divided pixels in each city cluster into five categories, including four urban categories and one rural category, based on each pixel's ISA, EVI, and their respective trends.

First, we determined the urban boundary for each city cluster and its surrounding rural areas. Urban boundaries were derived using the City Clustering Algorithm (CCA), which aggregates adjacent urban pixels (identified from the MODIS land cover data) into continuous clusters based on spatial proximity and land cover classification, providing an objective, data-driven definition of city extents independent of administrative boundaries. Specifically, we first identified urban pixels from the MODIS land cover product (classified as “urban and built-up” areas). The CCA then iteratively groups these urban pixels into clusters when the Euclidean distance between adjacent pixels is within one pixel resolution (with a larger value applied for cities divided by wide rivers), effectively connecting neighboring built-up pixels into cohesive urban clusters. This approach captures both core and peripheral urban areas, reflecting functional urban systems rather than fragmented built-up patches. The resulting spatially contiguous urban clusters define the urban boundary for each city. The entire process was implemented using the “cca” function in the osc library in R (version 4.1.2). To enable a consistent urban–rural comparison, we then delineated rural boundaries surrounding each city such that the total area of the selected rural region matched the total area of the corresponding urban footprint for that city. To avoid the disturbance of anthropogenic activities on natural vegetation change, we further restricted rural areas to pixels with an ISA value of 0 throughout the study period.

Second, we divided the urban areas within each city boundary into four dynamic categories based on the trends of ISA and EVI from 2003 to 2018. We first selected pixels in urban areas that fall into two ISA categories: urbanized areas (Ued), which have high ISA (>0.5 in 2003) and no increasing trend of ISA, urbanizing areas (Uing), which have low ISA (<0.5 in 2003) and an increasing ISA trend. These two categories represent 68.50% of the urban areas. We adopted ISA = 0.5 to distinguish urbanized from urbanizing areas, consistent with prior studies identifying similar threshold ranges (66, 67) and evidence that a 0.5 cutoff effectively separates built and nonbuilt surfaces (68, 69). We also conducted a sensitivity test using ISA = 0.4 and 0.6 and found that the proportion of classified categories is highly consistent, confirming the robustness of our results to threshold selection (Figs. S9 and S10).

Then we divided Ued and Uing into two subcategories based on EVI: greening (an increasing EVI trend) and browning (a decreasing EVI trend). We used the sign of the EVI or ISA slope rather than statistical significance to define trends, as our focus was on long-term directional patterns. This resulted in four urban dynamic categories: UedG, UedB, UingG, and UingB. These four categories constitute the focus study areas of our analysis and are collectively referred to as urban areas throughout the paper. Pixels that did not meet the criteria for any of the four urban categories were excluded from this analysis. Together with the rural category, they provide a comprehensive framework to assess the impact of dynamic interactions between urbanization and greenness changes in cities on UHI.

### ATC model

Remote sensing LST observations are frequently disrupted by cloud cover and precipitation, particularly in urban areas, resulting in substantial data gaps (70). To overcome this problem, we adopted an ATC model to statistically fit the expected LST time series based on available discrete remotely sensed LST observations. The ATC model omits day-specific anomalies, focusing instead on generating a climatological representation of long-term mean LST. This framework is advantageous for identifying gradual UHI evolution driven by urban expansion and vegetation change, while minimizing the influence of short-term weather disturbances. This model has been validated globally (53, 71) and across multiple satellite sensors (72), and it has been widely used to study urban thermal environment (28, 73, 74). Based on the ATC model, the annual cycle of temperature follows the seasonal progression of solar radiation and can be effectively modeled using the following sinusoidal function (53):


(1) $$\mathrm{LST}(d)=C_{m}+C_{a}\sin\;(2\pi \cdot \mathrm{DOY}/365-2\pi C_{p}/365+\pi /2),$$

where DOY is the day of year, and *C*_m_, *C*_a_, and *C*_p_ are three key parameters, representing the mean, amplitude, and the day with the largest value in the ATC.

We calculated *C*_m_, *C*_a_, and *C*_p_ in each pixel using the nlsLM function of library minpack.lm in R4.1.2 in two steps. We first fitted the ATC model using all the available LST data and calculated the absolute error between the fitted and observed LST. We found there are some extreme errors, due to the random extreme weather and cloud influence. We then calculated the threshold of an outlier (3/4quantile + 1.5IQR), excluded the observed data on these days (2.18%), and fitted the ATC model again. Moreover, to avoid the influence of extreme climate on the long-term LST change trend, we applied a moving average approach with a window of 3 years to calculate ATC parameters. Finally, we fitted the ATC in each pixel from 2003 to 2018.

The fitted LST was evaluated against the observed values in each city using four indicators, the correlation coefficient (*r*), mean error, root mean squared error (RMSE), and regression slope. Generally, the fitted LST showed strong agreement with observations in both annual and interannual variations (Fig. S11), with correlation coefficients larger than 0.9 in more than half of cities and a regression slope close to 1 in all cities (Fig. S12). The mean errors range from −2.11 to −1.01 °C, and RMSE ranges from 3.7 to 5.7 °C, consistent with the study of at the global (53) and local scales (74).

### Statistical analysis

Spatially, we calculated the difference of LST between urban and rural categories to quantify UHI intensity and its profile along four urban dynamic gradients. Similarly, we calculated the difference of EVI between urban and rural categories to quantify impacts of human activities on greenness (ie greening initiatives and urbanization). Temporally, we conducted linear regression analysis for all the variables from 2003 to 2018 and used the regression slope to represent their changing rate. Meanwhile, we calculated the mean values of UHI, ISA and EVI during 2003–2005 and during 2016–2018 to represent the initial and final periods to quantify their absolute temporal changes during the study period.

To attribute the contributions of urban expansion (ISA) and changes in greenness (EVI) to the change in UHI, we construct binary regression models, with UHI as the dependent variable and the urban–rural differences of imperviousness (ΔISA) and greenness (ΔEVI) as independent variables. To account for potential synergistic effects between urban expansion and greening, we also included an interaction term (ΔISA × EVI) in the regression model. These analyses were applied to the urbanizing regions (UingG and UingB), where both ISA and EVI exhibited substantial changes. In contrast, for the urbanized regions (UedG and UedB), ISA showed minimal temporal variation and no significant correlation with UHI (Fig. S8); therefore, only ΔEVI was included as the predictor in those models.

Given ΔISA and ΔEVI may have interaction in urbanizing areas, we tested for multicollinearity by calculating variance inflation factors (VIF) and examining correlation between ΔISA and ΔEVI (Table S1). All VIF values were below the standard threshold (VIF <3), indicating no multicollinearity. Correlations were low and insignificant for UingG areas, and moderately negative in UingB areas, reflecting the expected trade-off between increasing imperviousness and decreasing vegetation, rather than predictor dependency. Therefore, the regression models are considered robust in distinguishing the individual contributions of EVI and ISA to UHI change.

Given that ΔISA and ΔEVI exhibit different scales and ranges across urban dynamic categories within a city, we standardized both ΔISA and ΔEVI within four urban dynamic category for model fitting using z-score transformation as follows:


(3) $$\Delta \mathrm{IS}A_{z}=(\Delta \mathrm{ISA}-\mu_{\Delta \mathrm{ISA}})/\sigma_{\Delta \mathrm{ISA}\;}\;$$

(4) $$\Delta \mathrm{EV}I_{z}=(\Delta \mathrm{EVI}-\mu_{\mathrm{EVI}})/\sigma_{\Delta \mathrm{EVI}},$$

where *μ* and *σ* denote the mean and standard deviation of each predictor within four urban dynamic categories, respectively. This standardization enables comparison of effect sizes and model coefficients across groups by removing scale dependency.

Separate models were constructed for each city and more than 70% of models passed the significance test (*P*-values < 0.05). Using the regression models alongside changes in ISA and EVI over the study period, we calculated the UHI changes attributable to urban expansion (ΔUHI_ISA_ = *β*_ISA_*×*ΔISA*_z_*), vegetation greenness (ΔUHI_EVI_ = *β*_EVI_×ΔEVI*_z_*), and their interaction (ΔUHI_interaction_  *=*  *β*_interaction_*×*Δ(ISA*_z_* × EVI*_z_*). The modeled UHI change through the sum of ΔUHI_ISA_, fΔUHI_EVI_, and ΔUHI_interaction_ aligned well to observed UHI change (Fig. 4a), further suggesting the robustness of the established regression models.

## Supplementary Material

pgag070_Supplementary_Data

## Data Availability

All data used in this study are open-access and included in the paper and/or the Supplementary material. Daily LST data from MODIS product (MYD11A1) collection 6, monthly composite EVI data from MODIS product (MYD13A3) collection 6, and MODIS land cover type data (MCD12Q1) collection 6 at a resolution of 500 m in 2018 are available to download at https://search.earthdata.nasa.gov/. ISA data from annual maps of global artificial impervious area (GAIA) is available at https://data-starcloud.pcl.ac.cn/iearthdata/13. The scripts used to generate results are available at the GitHub: https://github.com/hdlimet/UHI_profile.
