# Supplementary material for: Will cities keep getting hotter? The interplay of urban expansion and greening reshapes future urban heat trajectories
Source: PNAS Nexus. 2026 Mar 16;5(3):pgag070. doi: 10.1093/pnasnexus/pgag070 (PMC13023365; doi:10.1093/pnasnexus/pgag070)
Supplement: pgag070_Supplementary_Data [file pgag070_supplementary_data.pdf]

Supplemental Material

Will cities keep getting hotter? The interplay of urban expansion and greening reshapes future urban heat trajectories

Huidong Li[1*], Lin Meng[1], Hiba Baroud[2], TC Chakraborty[3], Jiafu Mao[4], Zhonghua Zheng[5]

[1] Department of Earth and Environmental Sciences, Vanderbilt University, Nashville, TN, 37235, USA
[2] Department of Civil and Environmental Engineering, Vanderbilt University, Nashville, TN, 37235, USA
[3] Atmospheric Sciences and Global Change Division, Pacific Northwest National Laboratory, Richland, WA, 99352, USA
[4] Environmental Sciences Division, Oak Ridge National Laboratory, Oak Ridge, TN, USA
[5] Department of Earth and Environmental Sciences, The University of Manchester, Manchester, UK

[*] Corresponding Author: Huidong Li

Email: huidong.li@vanderbilt.edu

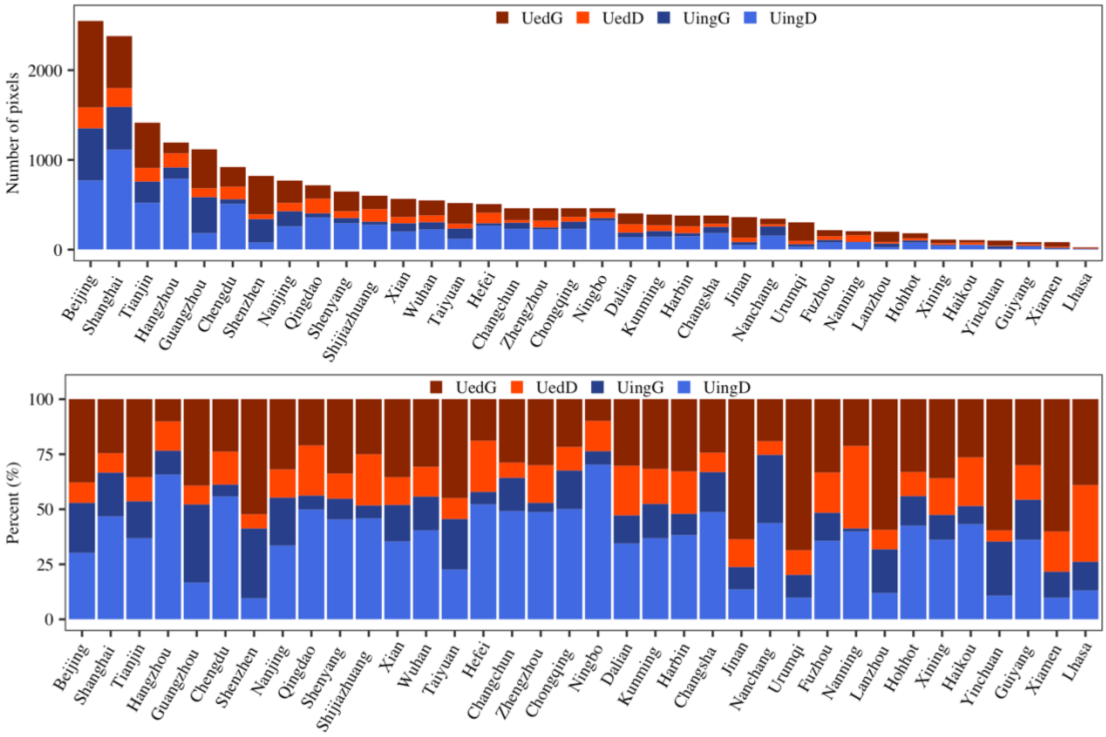

Fig. S1 Size and percentages of four urban dynamic categories in 36 study cities along with their city size.

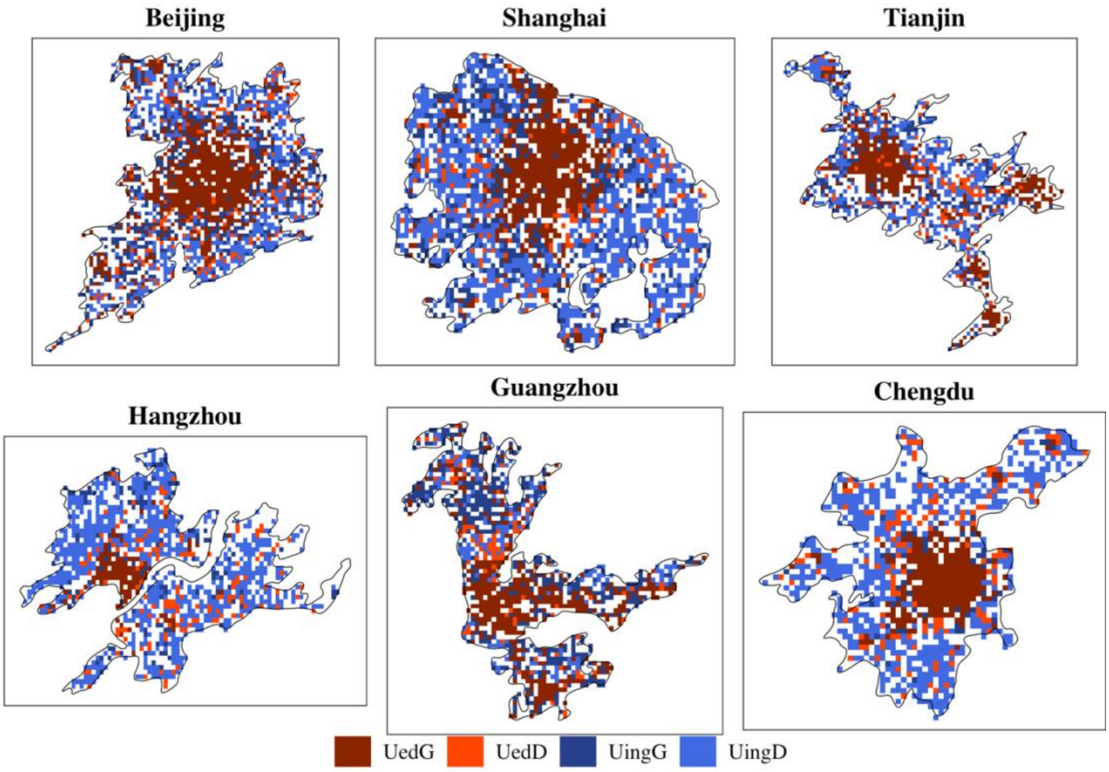

Fig. S2 Spatial pattern of four urban dynamic categories in the largest six cities. Urbanized-greening (UedG), urbanized-browning (UedB), urbanizing-greening (UingG), and urbanizing-browning (UingB).

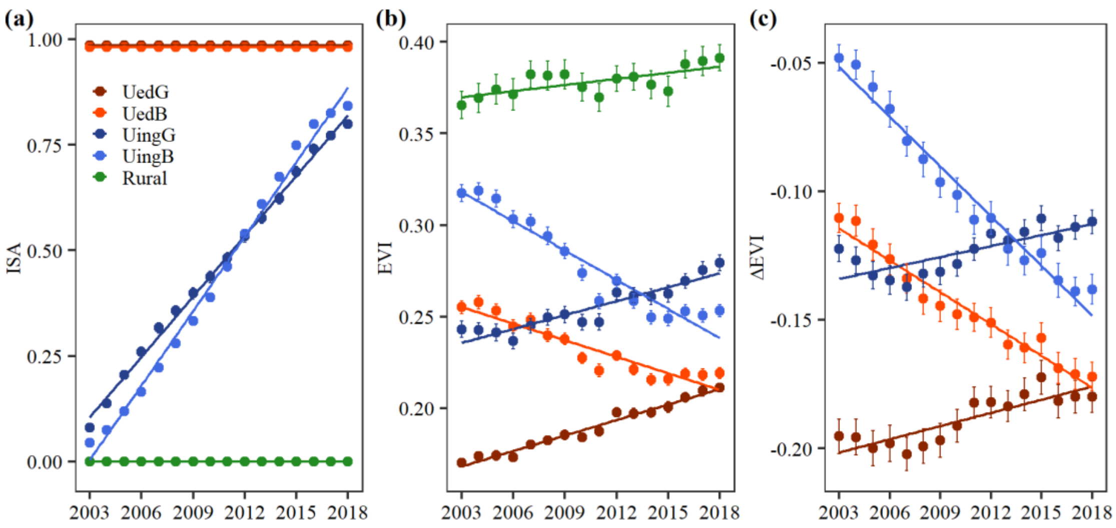

Fig. S3 Time series of imperviousness and greenness for different urban dynamic categories across all study cities during 2003-2018. (a): ISA; (b-c): EVI and its urban-rural difference. Error bar represents one tenth of the standard deviation among cities. The color represents different urban dynamic categories: urbanized-greening (UedG), urbanized-browning (UedB), urbanizing-greening (UingG), and urbanizing-browning (UingB).

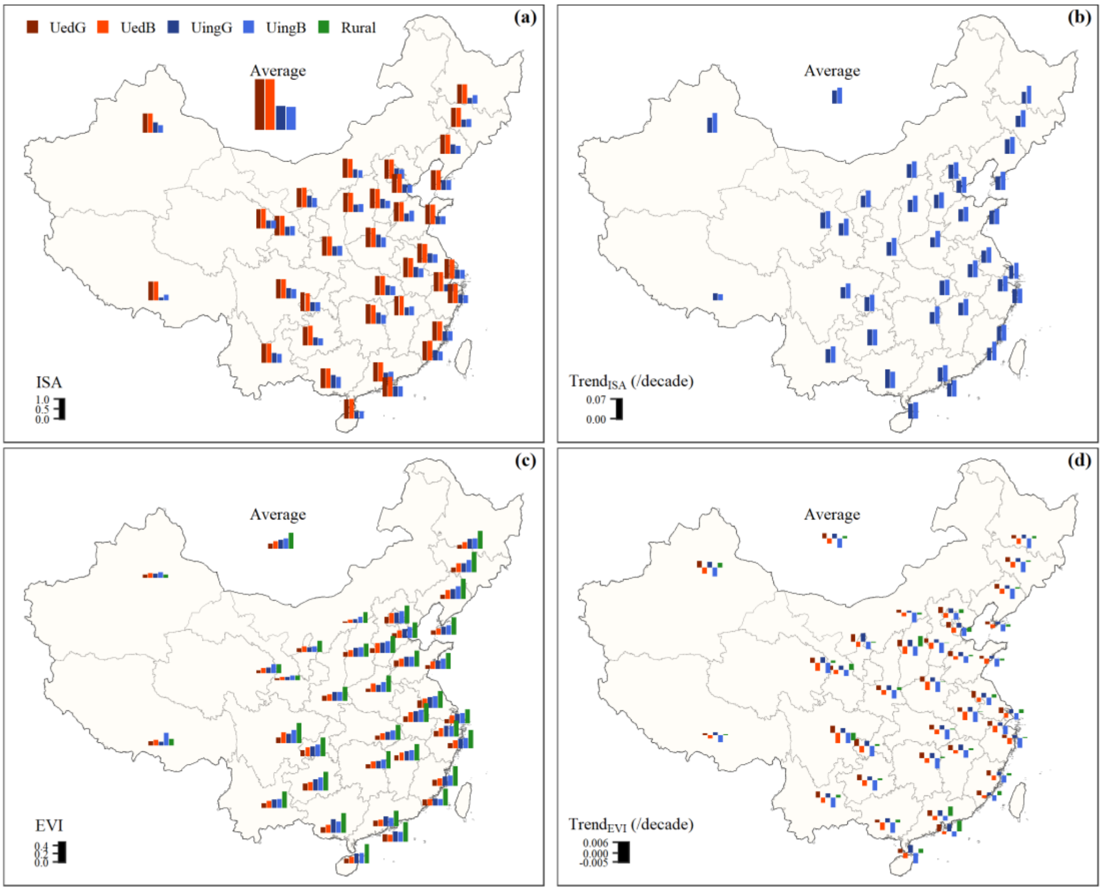

Fig. S4 Spatial patterns of ISA, EVI, and their trends from 2003 to 2018 across the 36 cities. (a-b): ISA and its trend; (c-d): EVI and its trends. The average values across all cities are shown at the top of each figure. The color represents different urban dynamic categories: urbanized-greening (UedG), urbanized-browning (UedB), urbanizing-greening (UingG), and urbanizing-browning (UingB).

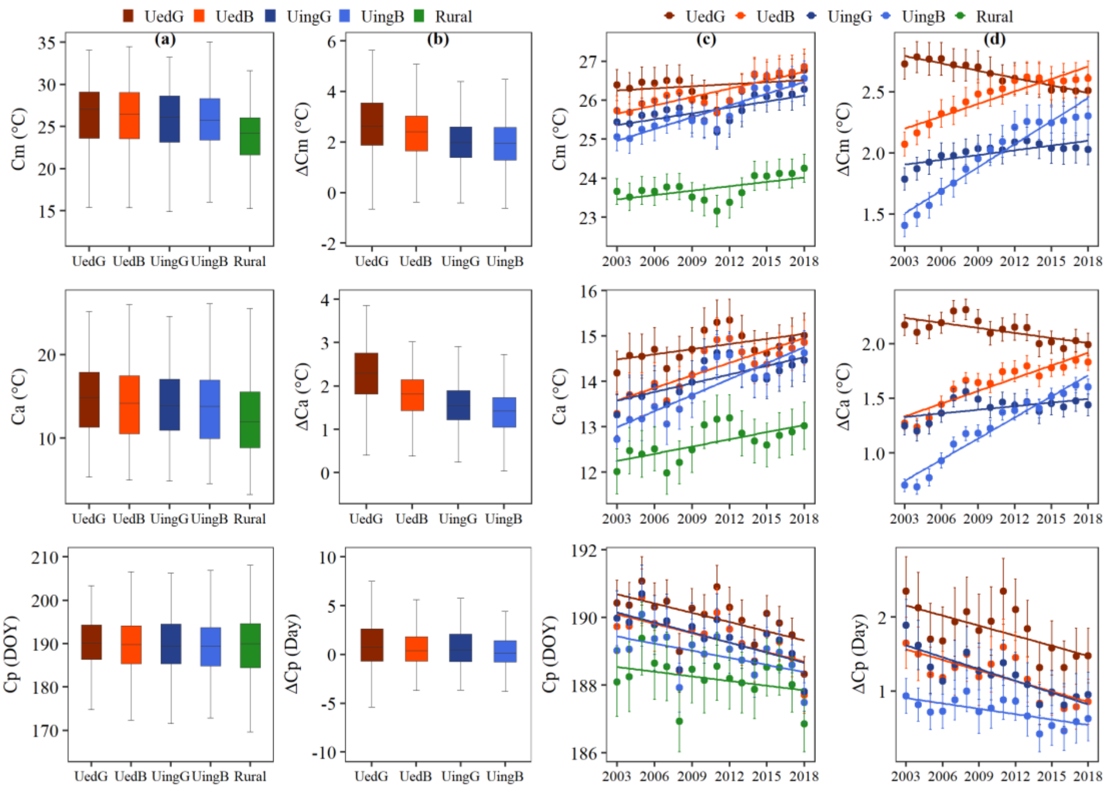

Fig. S5 Variations of annual temperature cycle parameters. (a-b): spatial variation and their urban-rural difference among urban dynamic categories. The middle line in each box plot represents the median value, while the box spans the interquartile range (IQR), capturing the middle 50% of the data. The whiskers extend to 1.5 times the IQR. (c-d): temporal variation and their urban-rural difference during 2003-2018. C_m: annual mean temperature; C_a: annual temperature difference between max and min; C_p: Julian day with the highest temperature. Δ represents the difference between urban categories and rural areas. Error bar represents one tenth of the standard deviation among cities.

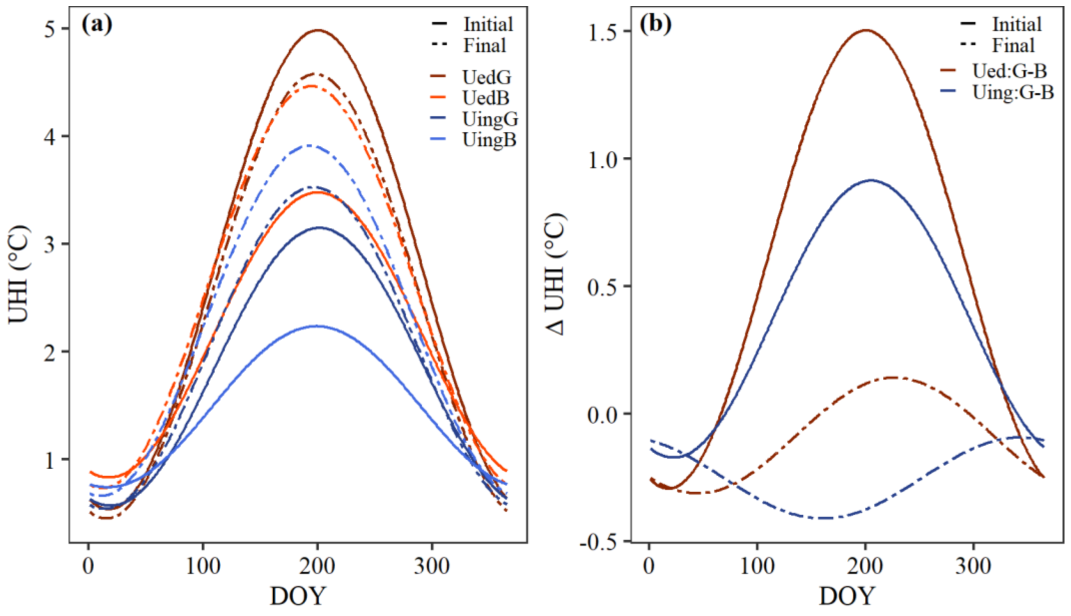

Fig. S6 Changes of annual UHI cycle between initial (2003-2015) and final (2016-2018) years. (a) changes for each urban category. (b) changes of the difference (ΔUHI) between greening and browning areas. ΔUHI was calculated by subtracting values in browning areas from greening areas. Urbanized-greening (UedG), urbanized-browning (UedB), urbanizing-greening (UingG), urbanizing-browning (UingB), UedG-UedB (Ued:G-B), and UingG-UingB (Uing:G-B).

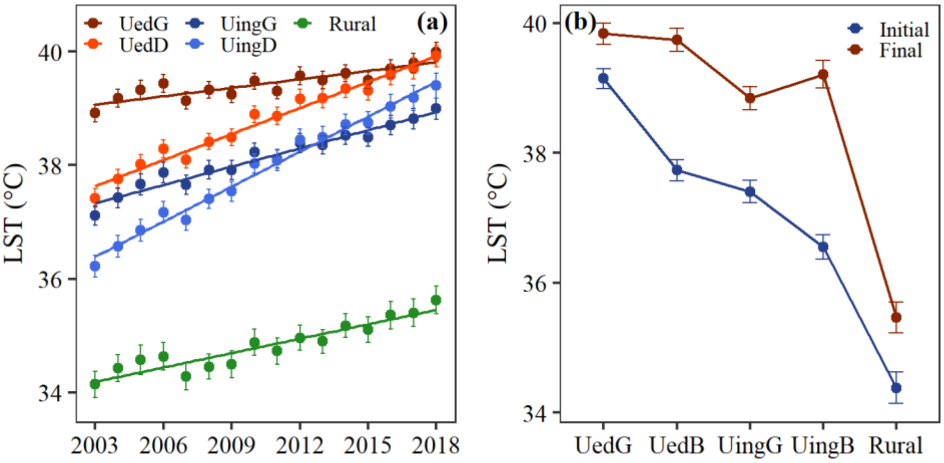

Fig. S7 Temporal variations of summer temperature along urban-rural gradient. (a): temporal change of temperature in urban and rural categories; (b): change in temperature profile between initial (2003-2015) and final (2016-2018) years. Error bars represent one tenth of the standard deviation among cities. Urbanized-greening (UedG), urbanized-browning (UedB), urbanizing-greening (UingG), and urbanizing-browning (UingB).

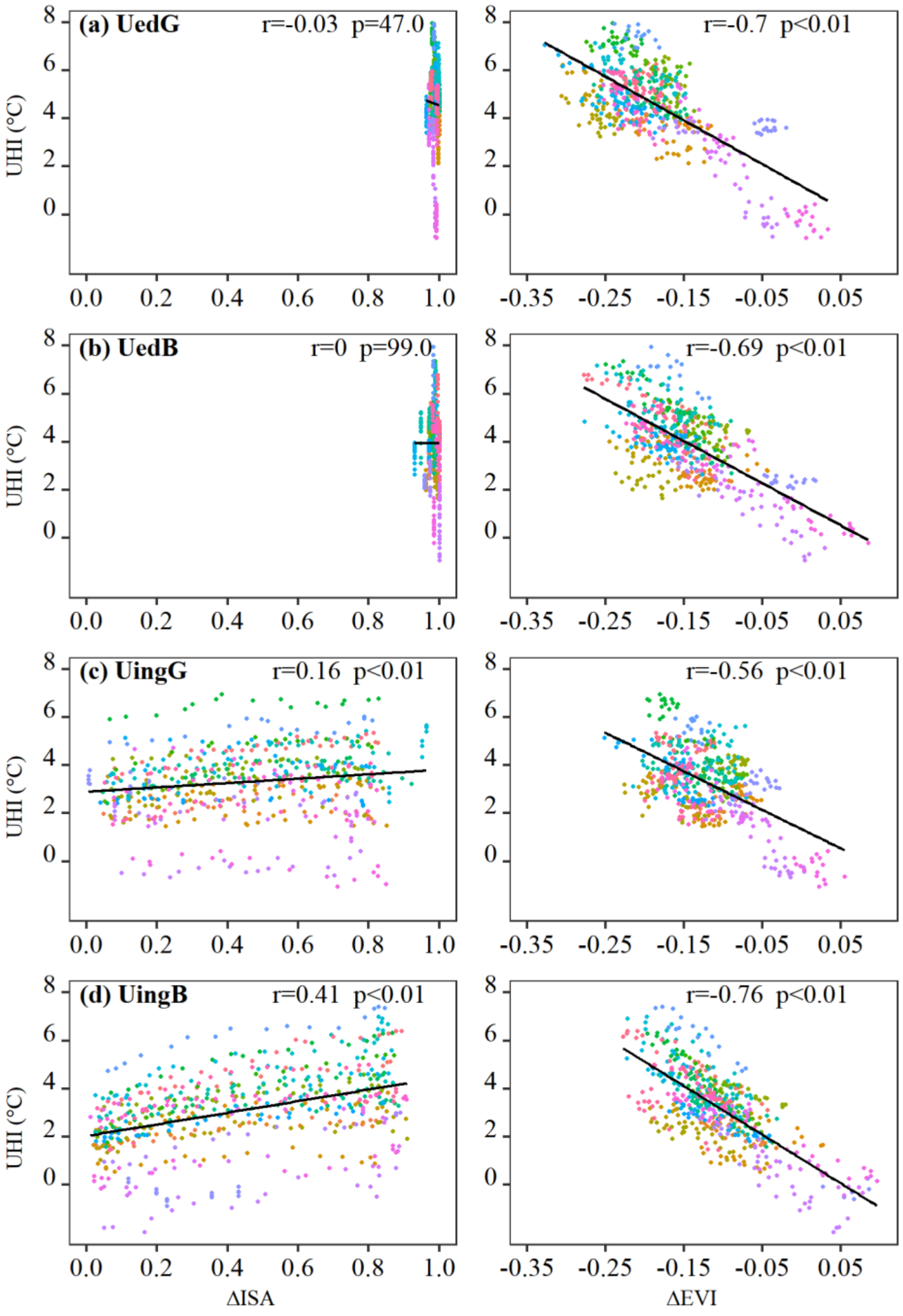

Fig. S8 Relationship between UHI and the urban-rural difference of ISA (left) and EVI (right) for four urban dynamic categories (a-d). Color represents different cities. Fitted linear regressions with correlation coefficient (r) and p-values are shown.

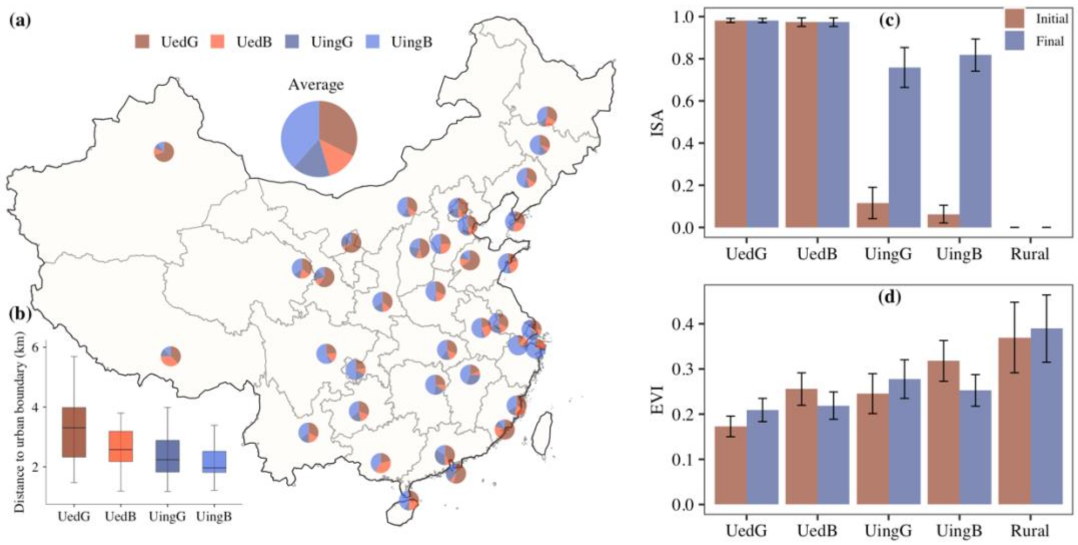

Fig. S9 Same as Fig. 1, but use the ISA=0.4 for urbanized and urbanizing classification. Divergent urban development pathways across all study cities. (a-b): Spatial pattern of area percentages of four urban dynamic categories in 36 cities and their distance to urban boundary. (c-d): Mean ISA and EVI between initial years (2003-2005) and final years (2016-2018). Error bar represents one standard deviation among cities. Urbanized-greening (UedG), urbanized-browning (UedB), urbanizing-greening (UingG), and urbanizing-browning (UingB).

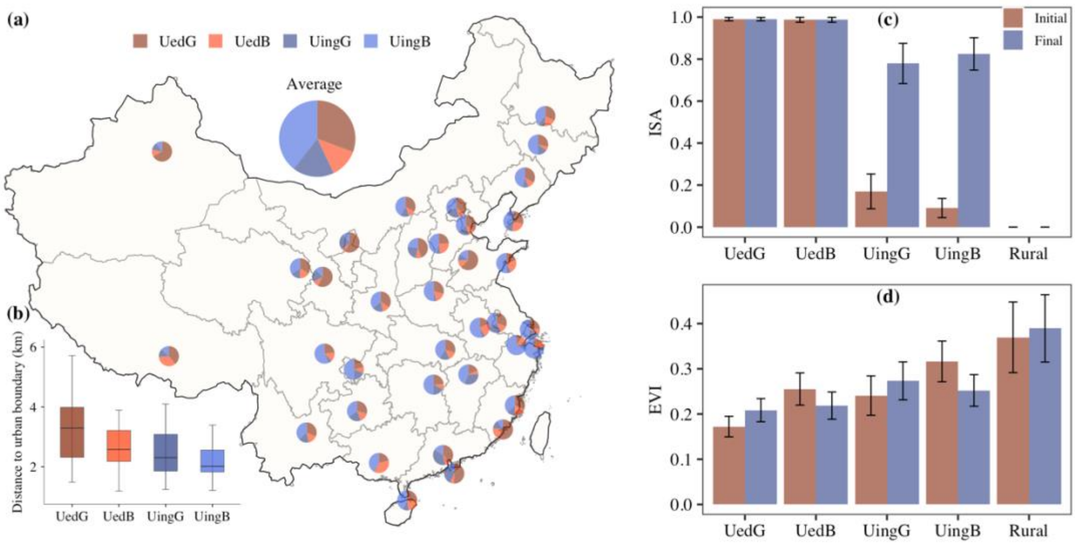

Fig. S10 Same as Fig. 1, but use the ISA=0.6 for urbanized and urbanizing classification. Divergent urban development pathways across all study cities. (a-b): Spatial pattern of area percentages of four urban dynamic categories in 36 cities and their distance to urban boundary. (c-d): Mean ISA and EVI between initial years (2003-2005) and final years (2016-2018). Error bar represents one standard deviation among cities. Urbanized-greening (UedG), urbanized-browning (UedB), urbanizing-greening (UingG), and urbanizing-browning (UingB).

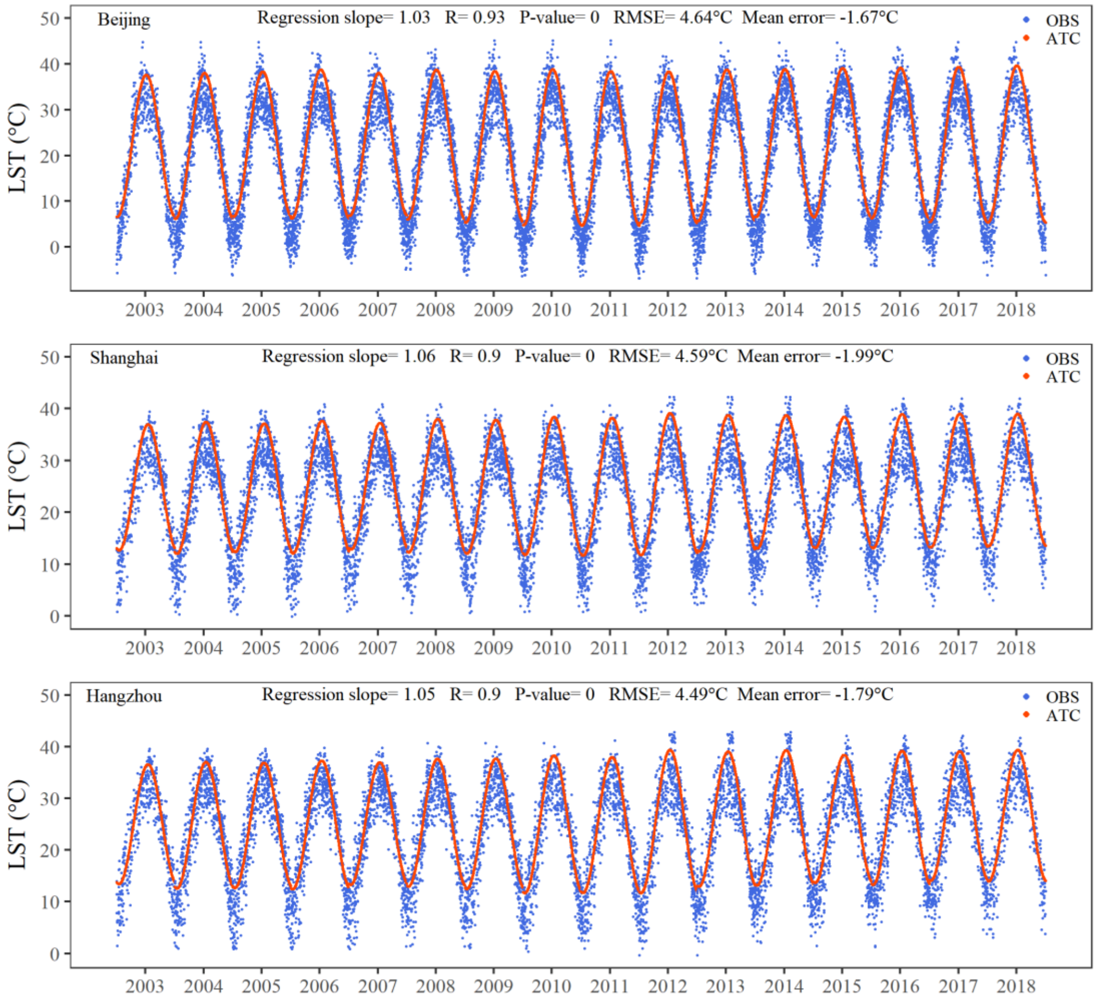

Fig. S11 Comparison of ATC model estimated and observed LST in three selected cities mentioned in the results (i.e. Beijing, Shanghai, and Hangzhou). Correlation coefficient (R), mean error, root mean squared error (RMSE), and regression slope are shown.

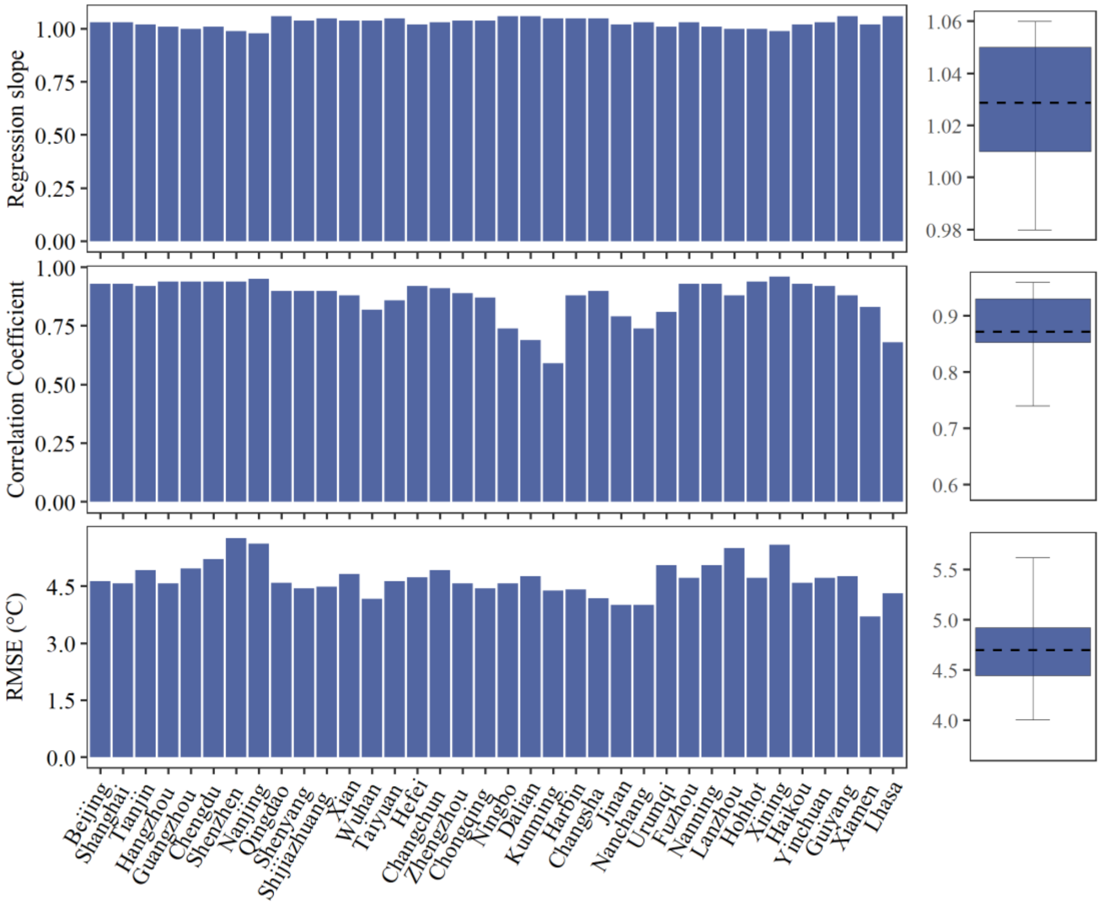

Fig. S12 The error matrix of the estimated temperature using the ATC model. Boxplot on the right shows the distribution of error matrices. The middle line in each box plot represents the median value, while the box spans the interquartile range (IQR), capturing the middle 50% of the data. The whiskers extend to 1.5 times the IQR.

Table S1. Correlation and variance inflation factor (VIF) between EVI and ISA in urbanizing regions across all study cities. UingG is urbanizing-greening, and UingB is urbanizing-browning.

Categories	Correlation		VIF
	Value	Significance	
UingG	0.02	P>0.05	1.00
UindD	-0.52	P<0.01	1.36
